# Hydrothermal Processing of Microorganisms: Mass Spectral Signals of Degraded Biosignatures for Life Detection on Icy Moons

**DOI:** 10.1021/acsearthspacechem.2c00213

**Published:** 2022-10-11

**Authors:** Tara L. Salter, Jonathan S. Watson, J. Hunter Waite, Mark A. Sephton

**Affiliations:** † Impacts and Astromaterials Research Centre, Department of Earth Science and Engineering, Imperial College London, London SW7 2AZ, United Kingdom; ‡ Space Science and Engineering Division, Southwest Research Institute, San Antonio, Texas 78238, United States

**Keywords:** astrobiology, icy moons, mass spectrometry, fatty acids, hydrothermal system, biosignatures, gas chromatography

## Abstract

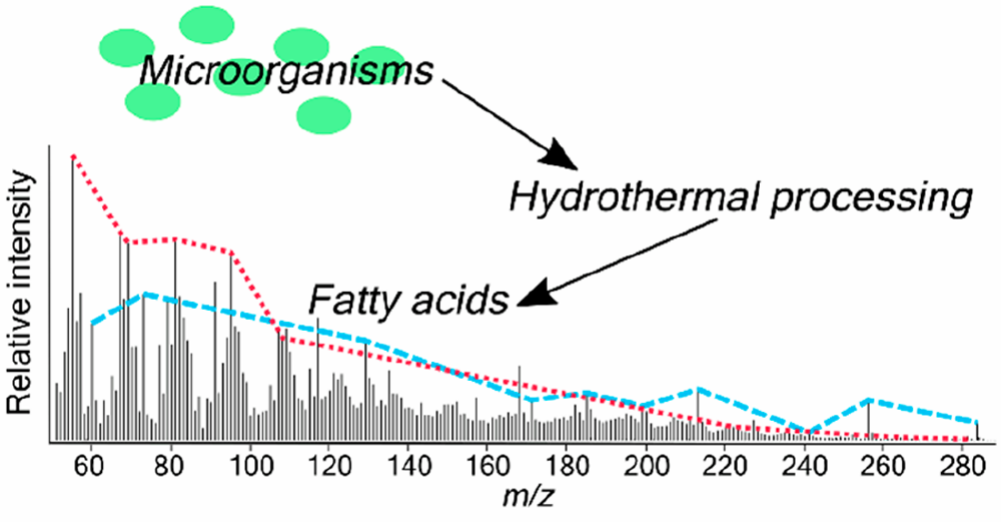

Life detection missions to the outer solar system are concentrating on the icy moons of Jupiter and Saturn and their inferred subsurface oceans. Access to evidence of habitability, and possibly even life, is facilitated by the ejection of subsurface material in plumes and outgassing fissures. Orbiting spacecraft can intersect the plume material or detect past sputtered remnants of outgassed products and analyze the contents using instruments such as mass spectrometers. Hydrothermalism has been proposed for the subsurface environments of icy moons, and the organic remains of any associated life would be expected to suffer some degradation through hydrothermalism, radiolysis, or spacecraft flyby impact fragmentation. Hydrothermalism is treated here for the first time in the context of the Europa Clipper mission. To assess the influence of hydrothermalism on the ability of orbiting mass spectrometers to detect degrading signals of life, we have subjected Earth microorganisms to laboratory hydrothermal processing. The processed microorganism samples were then analyzed using gas chromatography–mass spectrometry (GC–MS), and mass spectra were generated. Certain compound classes, such as carbohydrates and proteins, are significantly altered by hydrothermal processing, resulting in small one-ring and two-ring aromatic compounds such as indoles and phenols. However, lipid fragments, such as fatty acids, retain their fidelity, and their provenance is easily recognized as biological in origin. Our data indicate that mass spectrometry measurements in the plumes of icy moons, using instruments such as the MAss Spectrometer for Planetary Exploration (MASPEX) onboard the upcoming Europa Clipper mission, can reveal the presence of life even after significant degradation by hydrothermal processing has taken place.

## Introduction

The subsurface oceans of the icy moons of Jupiter and Saturn, notably those of Europa and Enceladus, may contain conditions amenable to life such as liquid water^1^ and chemical energy sources for metabolism.^2^ Plumes of material have been detected erupting from the tiger stripe fractures in the South Polar Terrain (SPT) of Enceladus,^3−6^ and similar plumes are thought to exist on Europa.^7−10^ These plumes can be used to sample the subsurface oceans of the icy moons by instruments onboard orbiting spacecraft.^11^ The ion and neutral mass spectrometer (INMS)^12^ onboard the Cassini-Huygens mission sampled material from the plumes of Enceladus and detected molecular hydrogen, interpreted as evidence for hydrothermal activity in the interior of the moon.^2^ Further evidence for hydrothermal processes come from the detection of nanometer sized silica grains in Saturn’s E ring.^13,14^ The detection of macromolecular organic material was also discovered by the INMS and the cosmic dust analyzer (CDA).^15^ The combination of a liquid water ocean with hydrothermal activity, and the detection of complex organic molecules, raises the question of whether the subsurface of Enceladus could have habitable conditions.

Water–rock interactions could form hydrothermal vents and drive prebiotic reactions. Here on Earth, one of the hypotheses for the origins of life is that it originated in hydrothermal systems.^16^ Present day hydrothermal systems are thought to be similar to the primordial conditions of early Earth and have been found to contain chemically reactive environments and to harbor rich ecosystems.^17^ Similar circumstances could arise in the subsurface oceans on the icy moons of Enceladus and Europa where hydrothermal vents have been postulated.^18,19^ If life does exist on icy moons, it will probably be as simple organisms, similar in complexity to Archaea and Bacteria found on Earth.^20^ It is also possible that these organisms, or their remains, may undergo alteration processes before being detected, with one possible alteration process being degradation in the hydrothermal systems that may be present on the icy moons. Other alteration processes that could degrade molecules on icy moons include radiolysis^21^ and impact fragmentation, as well as irradiation from UV and energetic ions and electrons.^22^ It is therefore important to understand how the molecular fingerprints of biology will be altered by hydrothermal processes and what remnants may persist of the biological signal in any processed residues; this will aid the chemical determination of habitability on icy moons. Spacecraft flying through the plumes of Enceladus and Europa may be able to detect the molecular fingerprints of intact and degraded life using mass spectrometry. It has been postulated that should there be life on Enceladus; it is possible that there could be between 10^5^ and 10^9^ cells cm^–3^ present in the hydrothermal vents on the seafloor of the icy moon.^23,24^ Bubble scrubbing may also increase the concentration of microorganisms in the plumes of Enceladus, with a possible plume density of 10^7^ cells cm^–3^.^24^

The upcoming NASA Europa Clipper mission will contain a next-generation mass spectrometer called the MAss Spectrometer for Planetary Exploration (MASPEX),^25,26^ as well as the Surface Dust Mass Analyzer (SUDA).^27^ MASPEX is a high mass resolution (>25 000 fwhm), high sensitivity (parts per million to parts per billion) time-of-flight mass spectrometer that will sample and analyze gaseous material. SUDA has a mass range of 1–250 Da and a mass resolution of 200–250 m/Δm.^27^

In this work, we have used hydrous pyrolysis to simulate the hydrothermal alteration of microorganism samples, with analysis of the subsequent products using gas chromatography–mass spectrometry (GC–MS). The data acquired from the experiments were used to generate simulated MASPEX mass spectra as may be encountered at Europa. Our experiments involve the heating of samples in a pressurized water environment up to ∼300 °C for periods up to 72 h. Hydrous pyrolysis has been used previously to examine the chemical processes of thermal maturation and aqueous processing within hydrothermal systems.^28,29^ The high temperatures and short duration of these experiments are analogous to the circulation of hydrothermal fluids through organic-rich sediments in vent systems resulting in the rapid thermal maturation of organic matter and have been used to study the synthesis of prebiotic organic compounds.^30^ Hydrous pyrolysis also produces results comparable to low temperature, long duration diagenesis^31^ and has previously been used for the artificial maturation of natural samples^32^ as well as studying the macromolecular material in meteorites.^33^ The hydrothermal processing of isoprenoid glycerol ether lipids from Archaea has previously been studied,^34,35^ with isoprenoid alcohols and hydrocarbons as the primary pyrolysis products formed. Similar results, i.e., the detection of isoprenoid alcohols and hydrocarbons, were also obtained from the hydrous pyrolysis of methanogenic Archaea.^36,37^ Hydrothermal simulation experiments have also been performed using autoclaves, with pressures and temperatures up to 600 bar and 600 °C, respectively.^38−40^ The autoclave setup is very similar to the hydrous pyrolysis system used in this study; however, when using an autoclave, material can be sampled while the experiment is running. Other hydrothermal reactor systems have been constructed that allow for the interaction between simulated hydrothermal fluids and ocean waters, at pressures of 100 bar and temperatures of 100–150 °C.^18,41^

In this study, we have used the cyanobacterium *Arthrospira* (*Spirulina*) and the alga *Chlorella* as model organisms to investigate the hydrothermal degradation mechanisms of the different cellular components: protein, lipid, and carbohydrate. To facilitate our experiments, we chose microorganisms with abundant lipid contents with the intention of recognizing fundamental changes that can be applied to chemical interpretations of data from a wider range of organisms. Hence, although cyanobacteria and algae may not be found in the conditions present on icy moons, they present lipid- and protein-rich microorganisms that are valuable as model samples. This work sought to understand the chemical changes experienced by microbes subjected to hydrothermal alteration; of particular interest was the lipids that are known from terrestrial experience to have diagnostic structures and relatively high preservation potential. We discuss the molecular fragments remaining after hydrous pyrolysis experiments at different temperatures and durations, generate simulated MASPEX mass spectra from the data, and discuss the implication for life detection on icy moons.

## Methods

### Samples

Cyanobacterium, *Arthrospira* (also known as *Spirulina*) and green alga *Chlorella* were purchased in powdered form from Whole Foods Market (Naturya brand, 100% microorganism with no additives). These samples were chosen to help understand the changes to the chemistry of the different components that make up the microorganisms, namely, the protein, lipid, and carbohydrate components, rather than understanding changes to the organism as a whole and the microbiology of the samples. Hence, in this respect, *Arthrospira* and *Chlorella* were used as lipid- and protein-rich model samples to understand these processes rather than using Archaea or Bacteria. Lipids are known to be the most preservable components of life even after hydrothermal alteration. We have previously characterized the pyrolysis–GC–MS response of a variety of archaea, bacteria, and algae.^42^

### Hydrous Pyrolysis

Hydrous pyrolysis was carried out following an adapted method by Sephton et al.^33^ Briefly, 15 mg of the powdered microorganism and 300 μL of degassed, deionized water were placed in a glass tube. The glass tube was then frozen in liquid nitrogen and flame-sealed under vacuum to ensure an inert atmosphere. Four glass tubes were placed inside a 75 mL 4740 stainless steel high pressure reactor (Parr Instrument Company) and heated according to the experimental conditions. Samples were subjected to temperatures of 200, 240, 270, or 300 °C. A range of temperatures was used to simulate conditions in different hydrothermal systems that have been found to have temperatures up to 350 °C.^43^ Experiments were carried out for 24 and 72 h to compare the differences between duration times; after 24 h, some reactions are completed, however after 72 h all reactions are thought to be complete, with all isomeric transformation reactions complete after that length of time.^44^ The pressure inside the vessel was dependent on the temperature, but for experiments at the maximum temperature of 300 °C, it was approximately 100 bar. The contents were left to cool to room temperature when the experiment was complete.

The heated contents were extracted from the glass tube with 500 μL of methanol (3×) and 500 μL of dichloromethane (DCM) (3×). All soluble matter was dissolved with this combination of solvents. The mixture was left to separate, and the DCM layer transferred into a clean test tube and the aqueous layer discarded. Sodium sulfate was used to remove any remaining water from the DCM extract. *N*,*O*-Bis(trimethylsilyl)trifluoroacetamide with 1% trimethylchlorosilane (BSTFA/TMCS) was added to produce the trimethylsilylated (TMS) derivatives, which are more amenable to analysis by gas chromatography–mass spectrometry (GC–MS). The derivatized extracts were then analyzed using GC–MS. Heating experiments were run in replicate. Experiments were also carried out with procedural blank control samples to check for contamination.

### Gas Chromatography–Mass Spectrometry Analysis

GC–MS analysis was carried out on a 7890 gas chromatograph coupled to a 5975 mass spectrometer, both Agilent Technologies. Separation was performed on a J&W DB-5 ms UI column (30 m in length, 0.25 mm internal diameter, and a film thickness of 0.25 μm). One microliter of solution was injected into the GC. The GC inlet was held at 270 °C and operated in split mode with a 10:1 ratio with a helium column flow rate of 1.1 mL min^–1^. The GC oven was held at 40 °C for 2 min and then increased to 310 °C at a rate of 5 °C min^–1^. The final temperature was held for 14 min. Mass spectra were acquired in electron impact mode (70 eV) with a scan range of *m/*z 45–550. To create mass spectra similar to what will be obtained by MASPEX, all the ions detected in a single experiment were summed over the relevant time range (9–50 min) of the total ion current and corrected to remove the TMS effects to produce a derivative-free spectrum. Peak identification was made by comparison with the NIST mass spectral library. Results are presented as qualitative data.

## Results

### Analysis of Nondegraded *Arthrospira* and *Chlorella* Samples

**Figure 1:**
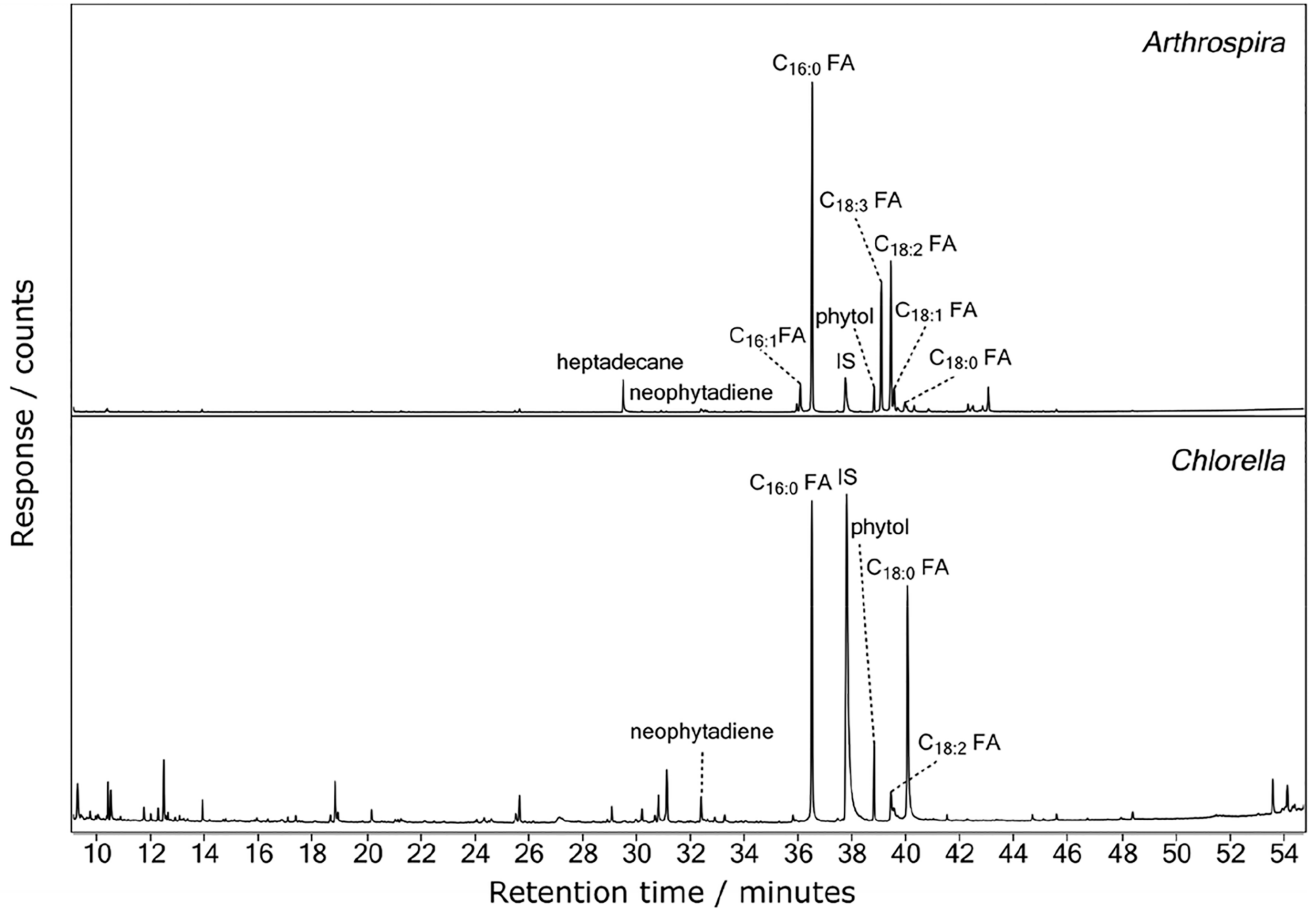
GC–MS total ion current (TIC) chromatograms of extracted and derivatized samples of unaltered *Arthrospira* (top panel) and *Chlorella* (bottom panel). FA, fatty acid; IS, internal standard.

Figure 1 shows the total ion current chromatograms for extracted and derivatized samples of *Arthrospira* and *Chlorella* that were not subjected to hydrothermal processing. Figure 1 is dominated by peaks from saturated and unsaturated fatty acids, with C_16:0_ being the most intense for both *Arthrospira* and *Chlorella*. Peaks are also observed for neophytadiene and phytol, which are decomposition products of chlorophyll^45^ and were also observed in the pyrolysis–GC–MS spectra of *Arthrospira* and *Chlorella*.^42^

### Hydrous Pyrolysis of *Arthrospira*

**Figure 2:**
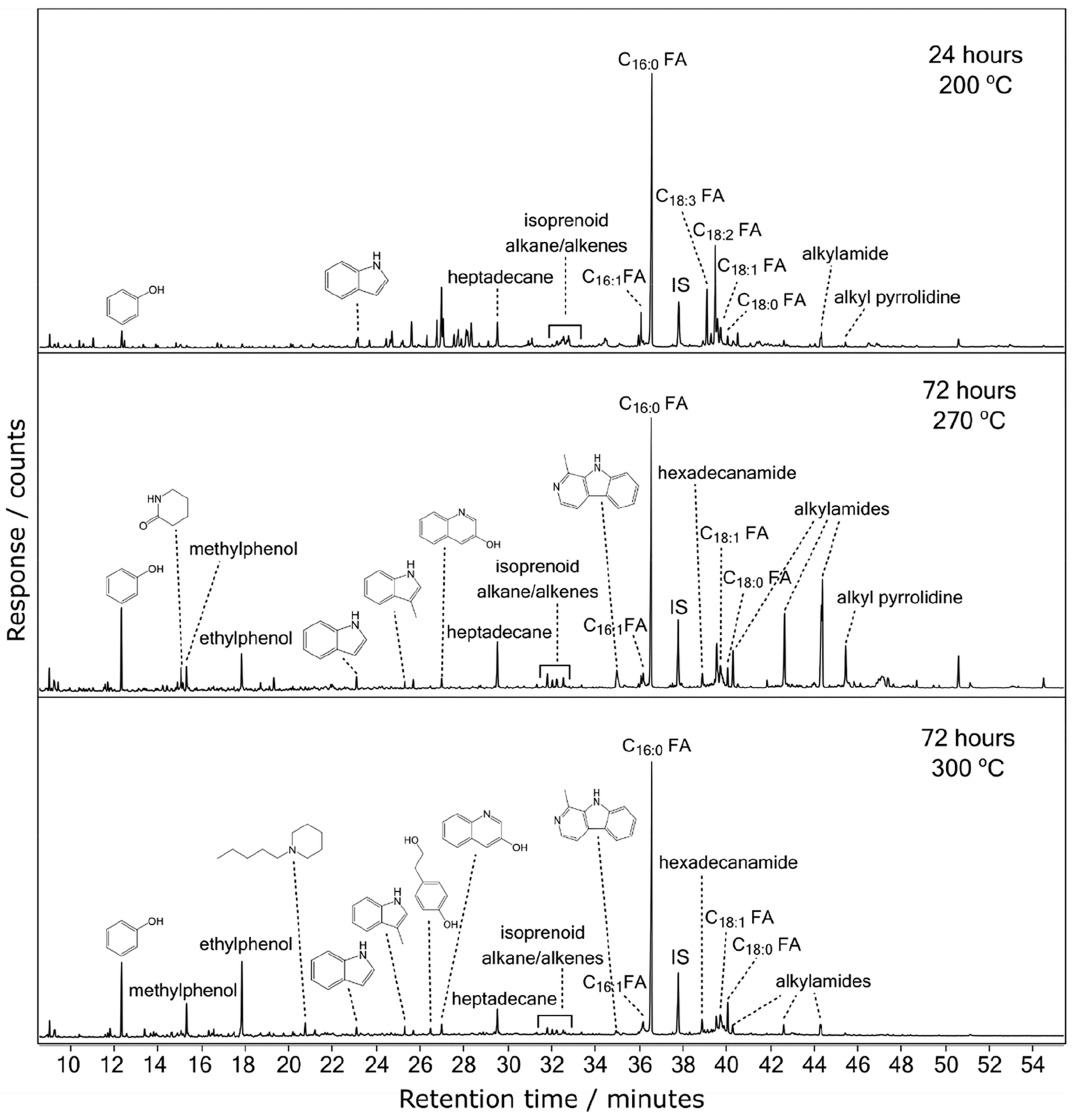
GC–MS total ion current (TIC) chromatograms of extracted and derivatized samples of *Arthrospira* subjected to hydrous pyrolysis for 24 h at 200 °C, 72 h at 270 °C, and 72 h at 300 °C (from top to bottom). FA, fatty acid; IS, internal standard.

*Arthrospira* and *Chlorella* were subjected to hydrothermal processing for 24 and 72 h at temperatures between 200 and 300 °C. Figure 2 shows the total ion current chromatograms from the GC–MS analysis of the extracted and derivatized products of hydrous pyrolysis of *Arthrospira* held for 24 h at 200 °C and 72 h at 270 and 300 °C. The top panel of Figure 2 shows the sample that was subjected to the least amount of processing, 24 h at 200 °C. A series of peaks, observed between 35 and 41 min, have been assigned to TMS derivatives of long chain fatty acids. The dominant peak observed in the total ion current was the C_16:0_ fatty acid. Other fatty acid peaks were assigned to saturated C_18:0_ fatty acid and unsaturated C_16:1_, C_18:1_, C_18:2_, and C_18:3_ fatty acids. In the lower retention time period, 10–30 min, low relative intensity peaks assigned to phenol, indole, and heptadecane were detected.

The two lower panels of Figure 2 show the extracted and derivatized products of hydrous pyrolysis of *Arthrospira* held for 72 h at 270 and 300 °C. These data, from the longer experiment durations and higher temperatures, show several differences to that of the data for 200 °C held for 24 h. First, the relative intensity of the unsaturated fatty acids, C_16:1_, C_18:3_, and C_18:2_, decreased as the sample processing increased. However, the saturated fatty acids, C_16:0_ and C_18:0_, do not decrease in relative intensity. For the longer duration experiments, a series of smaller molecular fragmentation products was observed. These have been assigned to aromatic compounds containing oxygen and/or nitrogen atoms, e.g., indole, phenol, and their derivatives. The intensity of the isoprenoids, phytanol, phytane, and phytene remained relatively unchanged by the increased sample processing. Heptadecane increased in relative intensity in the 270 °C data but decreased again in the 300 °C data. For the 270 °C data, peaks assigned to alkylamides and alkyl pyrrolidine (alkylpyridine) increased in intensity. However, the alkylamides and alkyl pyrrolidine peaks decreased in intensity in the 300 °C data. Other minor changes, such as a small increase in the relative intensity of methylphenol (cresol) and ethylphenol, were observed between the total ion currents for the experiments held for 72 h at 270 and 300 °C.

As well as the data shown in Figure 2, additional data were obtained for hydrous pyrolysis experiments held for 24 h at 240, 270, and 300 °C and for experiments held for 72 h at 200 and 240 °C. Minimal qualitative changes to the total ion currents were observed between the 24 h data for 200 and 240 °C and also for the 200 °C data for 24 and 72 h. The data for 270 and 300 °C were also similar for both 24 and 72 h experiments; hence, for conciseness, only the data showing major changes are displayed in Figure 2.

Several differences were observed between the nondegraded *Arthrospira* samples and the degraded samples (Figures 1 and 2). First, neophytadiene and phytol, which were present in the nondegraded data, are absent in the samples that underwent hydrous pyrolysis. Second, alkylamides and degradation products of proteins and carbohydrates, such as phenols and indoles, are absent in the nondegraded data, however they are present in the hydrous pyrolysis samples. Fatty acids, particularly C_16:0_, are the dominant peaks in both the nondegraded and degraded data. However, a higher relative proportion of unsaturated fatty acids, such as C_18:3_ and C_18:2_, are detected in the nondegraded data. These are subsequently degraded in the samples that have undergone hydrous pyrolysis. The saturated fatty acids, C_16:0_ and C_18:0_, are the most recalcitrant.

### Hydrous Pyrolysis of *Chlorella*

**Figure 3:**
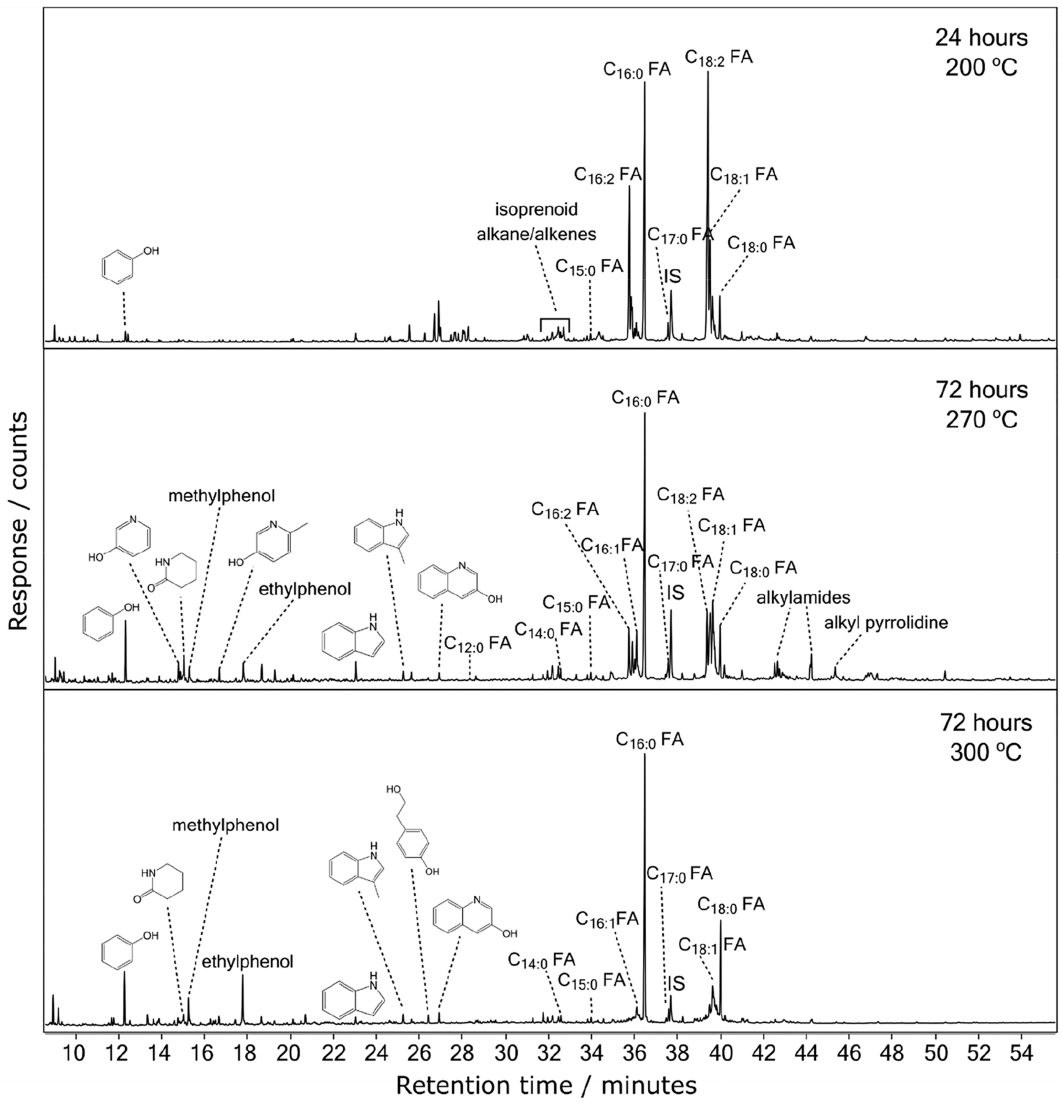
GC–MS total ion current (TIC) chromatograms of extracted and derivatized samples of *Chlorella* subjected to hydrous pyrolysis for 24 h at 200 °C, 72 h at 270 °C, and 72 h at 300 °C (from top to bottom). FA, fatty acid; IS, internal standard.

Hydrous pyrolysis of *Chlorella* was carried out under the same experimental conditions as *Arthrospira*. Figure 3 shows the total ion current chromatograms from the GC–MS analysis for the extracted and derivatized hydrous pyrolysis products of *Chlorella* held at 200 °C for 24 h and 270 and 300 °C held for 72 h. There are notable differences to the *Arthrospira* data. First, the *Chlorella* data with the lowest amount of sample processing, 200 °C held for 24 h, shown in the top panel of Figure 3, has a large relative intensity for the unsaturated fatty acids C_16:1_, C_16:2_, C_18:1_, and C_18:2_, when compared to the data for *Arthrospira* under the same experimental conditions. The relative intensity of the unsaturated fatty acids decreased as the sample processing increased; in accordance with a similar trend also observed in the *Arthrospira* data. Small relative intensity peaks of saturated C_14_, C_15_, and C_17_ fatty acids were also observed in the *Chlorella* data. The small molecule fragmentation products, indole, phenol, and their derivatives, detected in the *Arthrospira* hydrous pyrolysis experiments were also observed for the *Chlorella* data as the sample processing increased and are shown in the lower two panels of Figure 3. Another noticeable difference was the absence of heptadecane in the *Chlorella* data. Alkylamide and alkylpyridine peaks were also observed in the *Chlorella* data at 270 °C; however, their relative intensities were much lower than the same peaks in the *Arthrospira* data (Figure 2). Similar to the *Arthrospira* data, the saturated fatty acids, C_16:0_ and C_18:0_, are the most recalcitrant and do not show any signs of decay under the longest duration and highest temperature conditions.

Similarly, to *Arthrospira*, the same differences were observed between the *Chlorella* samples that were nondegraded and those that have undergone hydrous pyrolysis. Namely, in the nondegraded data, neophytadiene and phytol are present whereas alkylamides, phenols and indoles are absent (Figure 1). However, the fatty acid distributions are different, with a lower relative proportion of unsaturated fatty acids detected in the nondegraded *Chlorella* samples compared to the degraded samples. The opposite is observed for *Arthrospira*, where the nondegraded data show a higher relative proportion of unsaturated fatty acids compared to the degraded data.

## Discussion

### Mechanisms of Degradation

Hydrous pyrolysis reveals the differences in the hydrothermal stability of the different components of microorganisms, and similar trends are observed for both *Arthrospira* and *Chlorella*. However, *Chlorella* is an alga and *Arthrospira* is a cyanobacterium and they have slightly different starting compositions: *Arthrospira* is typically 46–63% protein, 8–14% carbohydrate, and 4–9% lipid (dry weight), whereas *Chlorella* has a greater proportion of carbohydrates and lipids, 12–17 and 14–22%, respectively.^46^*Chlorella* is a spherical unicellular organism, whereas *Arthrospira* is a filamentous, spiral-shaped multicellular organism. Table 1 lists the types of molecules detected from the hydrous pyrolysis of *Arthrospira* and *Chlorella* and their possible origin. The comparison between the results from the two strains gives insight into the degradation mechanisms occurring in hydrous pyrolysis (these are detailed below). The compounds detected with the largest signal intensities in the results presented here are the lipid degradation products, fatty acids, formed from hydrolysis of lipids. In the hydrous pyrolysis data for both *Chlorella* and *Arthrospira*, saturated fatty acids (C_16:0_ and C_18:0_) appear to be the most resistant to degradation. However, all the other molecules detected reveal evidence of degradation relative to the saturated fatty acids. This is indicated in the reduction in signal intensity of the unsaturated fatty acids and also the increase in small fragment molecules, such as phenol and its derivatives (Figures 2 and 3). The degradation of unsaturated fatty acids is most clearly observed in the *Chlorella* data between the least and most amount of sample processing where the C_18:2_ peak reduced dramatically, as shown in the top and bottom panels of Figure 3.

**Table 1:** Molecules Detected from the GC–MS Analysis of Hydrous Pyrolysis of *Arthrospira* and *Chlorella* and Their Characteristic Fragment Ions and Possible Origin

original microorganism component	hydrous pyrolysis product	characteristic fragment ions (*m*/*z*)	references
lipid	saturated fatty acids	60, 73, 129, 171, 185, 199, 213, 241, 256, 284	(47)
	unsaturated fatty acids	55, 67, 69, 79, 81, 83, 93, 95, 97, 108, 110, 222, 236, 264, 280, 282	
	*n*-alkanes	57, 71, 85, 99, 113	
	alkylamides	57, 59, 72, 73, 115, 128, 129, 142	(53, 56, and 57)
	alkyl pyrrolidine	98, 113, 126	
protein	indoles	90, 117, 130, 154, 182	(67)
	phenols	57, 66, 77, 94, 107, 108, 122, 191	
	piperidinones	55, 70, 99	
	pyridinols	80, 95, 109	
	alkylamides	57, 59, 72, 73, 115, 128, 129, 142	(53, 56, and 57)
carbohydrate	phenols	57, 66, 77, 94, 107, 108, 122, 191	(53)
chlorophyll	isoprenoid alkanes/alkenes	55, 57, 70, 71, 83, 85, 97, 99, 111, 113, 125, 127, 140	(45)

The higher thermal stability of lipid fragments, compared to protein and carbohydrates, is well established in the literature.^47^ Results from the hydrous pyrolysis of a cyanobacterial-dominated microbial mat showed that, at lower temperatures, a larger proportion of carbohydrate biomolecular peaks were detected; however, as the temperature was increased, lipids became the dominant simulated geomolecules.^48^ The destruction of unsaturated fatty acids at higher temperature hydrous pyrolysis conditions has also been observed previously.^49^

As detailed above, small molecular fragments, phenols and nitrogen heterocycles, were detected as the amount of sample processing increased. Nitrogen heterocycles, such as pyrroles, indole, and indole derivatives, are produced from the breakdown of proteins. The peptide bond rapidly hydrolyses under hydrothermal conditions, and amino acids are known to subsequently degrade through decarboxylation and deamination.^50^ Nitrogen containing heterocycles (pyrroles and pyridines) are produced from the Maillard reaction between amino acids and reducing sugars, which in turn are formed from the hydrolysis of protein and carbohydrate components.^51,52^ Phenol, and its derivatives, can be formed from the fragmentation of proteins and carbohydrates.^53,54^ However, no other breakdown products of carbohydrates are observed in our experiments (Figures 2 and 3), although this may be due to the extraction method used, which only observes the hydrophobic products, as well as the low proportion of carbohydrates in the microorganisms analyzed. This is in agreement with the pyrolysis–GC–MS of *Chlorella* and *Arthrospira* that only show very small amounts of carbohydrate fragmentation products.^42^ Phenols and nitrogen heterocycles were also detected in the pyrolysis–GC–MS of archaea and bacteria and are good indicators of breakdown products of proteins.^42^ The increase in relative intensity of phenol and nitrogen heterocycles as the temperature and duration of the hydrous pyrolysis experiments increased is another indicator of the degradation of the microorganisms into smaller molecular fragments. Modeling of the decomposition rates of amino acids in water, relevant to oceans on icy moons, reveals that they decompose over relatively short geological time scales (<1 Ma) in a hydrothermally active ocean.^55^

Peaks corresponding to alkylamides are detected in all the experimental conditions; however, their maximum is achieved under conditions of 270 °C held for 72 h, and they are much more abundant in *Arthrospira* than *Chlorella*. Alkylamide formation is the result of condensation reactions between lipid and protein components in the cyanobacteria. Specifically, the protein breaks down into amino acids and subsequently ammonia, which then goes on to react with the fatty acids, forming alkylamides.^53,56,57^ At the highest processing conditions, 300 °C held for 72 h, the relative intensity of alkylamides decreased; Gai et al.^53^ also noted this decrease in alkylamides as the temperature increased. Hydrous pyrolysis experiments of the fatty acid, *n*-nonadecanoic acid with ammonium bicarbonate have been shown to produce an array of amides, alkyl nitriles, and *N*-methylalkyl amides.^58^ The very low intensity of alkylamides in *Chlorella* has been reported previously and is thought to be due to competing reactions between the degradation products of carbohydrates and proteins.^59^

From the data presented in Figures 2 and 3, we observed that the relative intensities of the isoprenoids do not change with different hydrothermal processing conditions. The relative intensity of heptadecane reached a maximum in the data for 270 °C held for 72 h. The observation of *n*-C_17_ alkane is typical of lipids from cyanobacteria.^60,61^ This is also consistent with the absence of significant amounts of *n*-C_17_ alkane in the *Chlorella* samples in the present study.

### Preservation of Lipid Biosignatures

We have shown above that part of the biotic fingerprint of the microorganisms, namely, fatty acids, were retained even under the most extreme hydrothermal conditions used in this study, at 300 °C held for 72 h. Although lipid structures are degraded, characteristic straight-chain saturated fatty acids from lipid fragmentation are thermally stable and are preserved in the conditions used in this study. Small molecular fragments, such as phenols and indoles, from proteins and carbohydrates are also detected and reveal that hydrothermalism of microbial biomass can generate one-ring and two-ring aromatic units at the lowest temperatures of our study and their relative intensity increases with harsher degradation conditions.

Abiotic materials can contain fatty acids, and they can also be generated abiotically under hydrothermal conditions; however, this produces a range of lipid species with no carbon number preference.^30^ In this work, we only detect discrete fatty acid molecules of a particular chain length, e.g., C_16:0_ and C_18:0_, which are characteristic of biotic signals, rather than a smooth distribution of molecules dependent on chemical properties alone,^62,63^ which is characteristic of abiotic signals. In agreement with the results presented here, C_16:0_ is the most abundant biologically produced long chain fatty acid detected in nature.^64^

Cold and salt adapted extremophiles, as well as methanogens, are more likely than cyanobacteria to be present on icy moons.^20^ However, *Arthrospira* and *Chlorella* were selected for use in this study as they provide good model organisms and we can use the dominant trends in this work to extend our results to other strains of archaea and bacteria, such as those analyzed previously.^42^ Differing amounts of the molecular products of protein, carbohydrate, and lipid fragmentation were detected from several extremophile strains of archaea and bacteria using pyrolysis–GC–MS. From our work discussed above, we know that protein and carbohydrate structures are degraded to small molecules such as the one-ring and two-ring aromatic compounds phenol and indole and that only the lipid fragmentation fingerprint is preserved. Therefore, we would expect similar mechanisms to apply to other types of archaea and bacteria although they may have differing initial compositions. While proteins and carbohydrates are common components of all microorganisms, variations in the decomposition products may be observed if the original microorganisms have different molecular constitutions.

### Generation of MASPEX Simulated Mass Spectra

**Figure 4:**
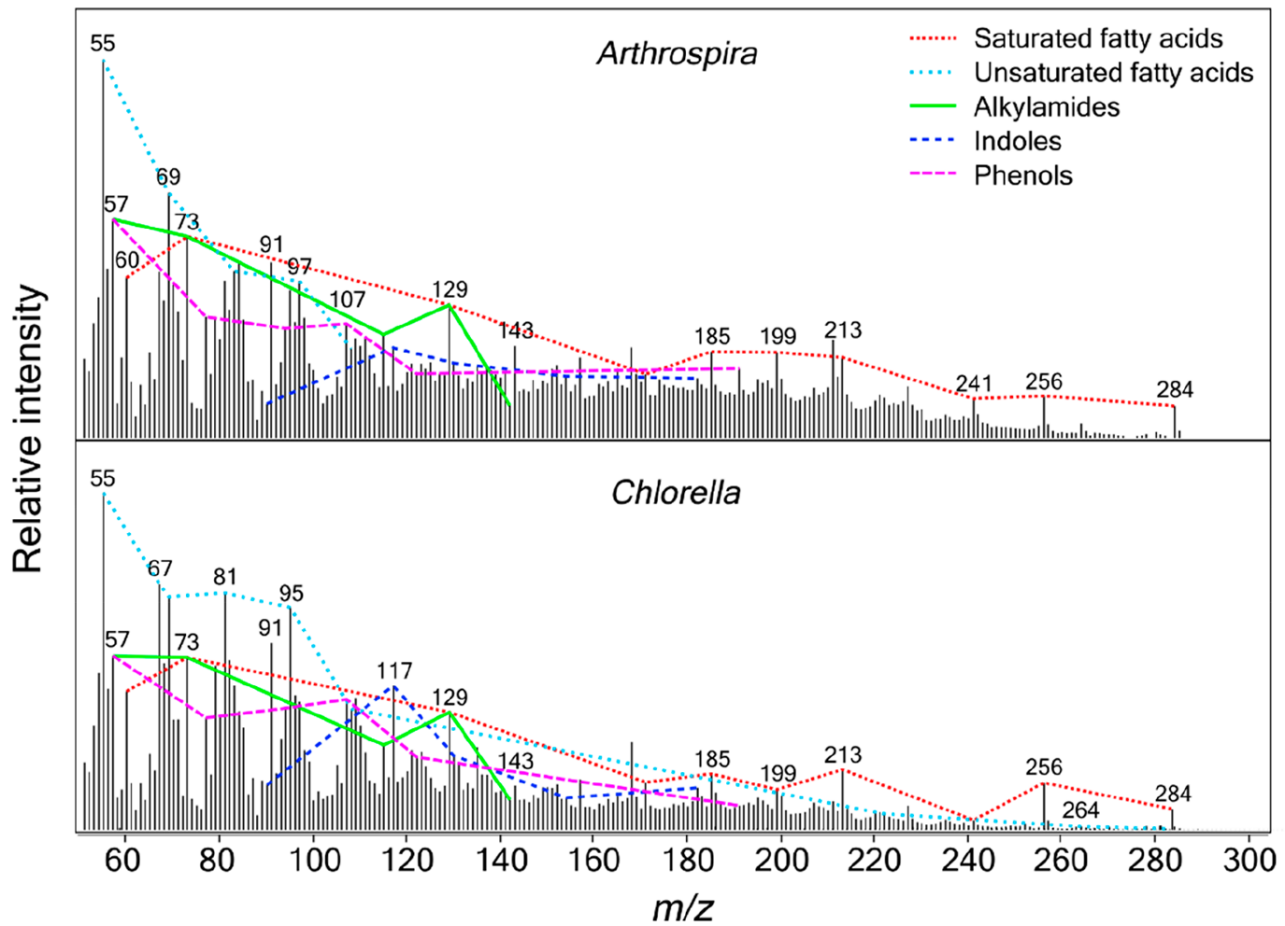
MASPEX simulated mass spectra of *Arthrospira* and *Chlorella*, after hydrous pyrolysis at 300 °C for 72 h. Although derived from TMS derivatives, the mass spectra have been corrected to present data that is comparable to that expected by a MASPEX type instrument. Ions from the same compound family are linked by lines, labeled according to the legend.

The results presented in this study were obtained using GC–MS with unit mass resolution; however, accurate identification of molecules was possible due to the gas chromatography separation technique. The mass spectrometers onboard missions to icy moons are currently not coupled to separation techniques. Instead, MASPEX, which will be onboard the upcoming Europa Clipper mission, has high mass resolution capability, up to 25,000 fwhm, enabling it to identify ions with greater precision.^25^ To produce results from the data acquired in this study that are comparable with MASPEX, mass spectra have been generated from total ion currents. This method has previously been used to generate mass spectra from pyrolysis–GC–MS data, which showed the ion series from different fragmentation products.^42^Figure 4 shows the mass spectra for *Arthrospira* and *Chlorella* generated from the total ion currents for the hydrous pyrolysis experiment at 300 °C held for 72 h. As the hydrous pyrolysis extracts were derivatized to make them easier to analyze using GC–MS, many of the molecules were detected as TMS derivatives. Therefore, the mass spectra obtained from the total ion currents in this study were modified, using subtraction, to remove the TMS derivatives and produce a MASPEX-like spectrum.

The mass spectra in Figure 4 show the ions detected from the different components of the degraded microorganisms, corrected to remove the TMS derivatives. MASPEX will be able to acquire data up to approximately *m*/*z* 600.^25^ The data in this study were acquired in the mass range *m*/*z* 45–550; however, in Figure 4, only peaks up to *m*/*z* 300 are displayed due to the very low intensity of peaks detected above this range. The fragmentation patterns of individual molecules that are indicative of protein, carbohydrate, and lipid structures have been determined using GC–MS, and in Figure 4, ions from the same compound family have been linked together by lines. For simplicity, only the most abundant ions have been marked on the spectra. The mass spectra are dominated by peaks from fatty acids: e.g., *m*/*z* 73, 129, 185, 256, 284. Similarities are observed between *Arthrospira* and *Chlorella*, such as the detection of fatty acids. The characteristic ions detected from the different molecular types have been added to Table 1. The mass spectra produced in this study add to the mass spectral library of fragmentation patterns generated from the pyrolysis–GC–MS of bacteria and archaea^42^ and extend that work to signals of microbial life affected by hydrothermal degradation. These ion series greatly help with the interpretation of complex mass spectra when no separation technique is present. These data can be used as a reference for future data obtained by MASPEX, enabling the determination of biological fingerprints or their degraded counterparts.

**Figure 5:**
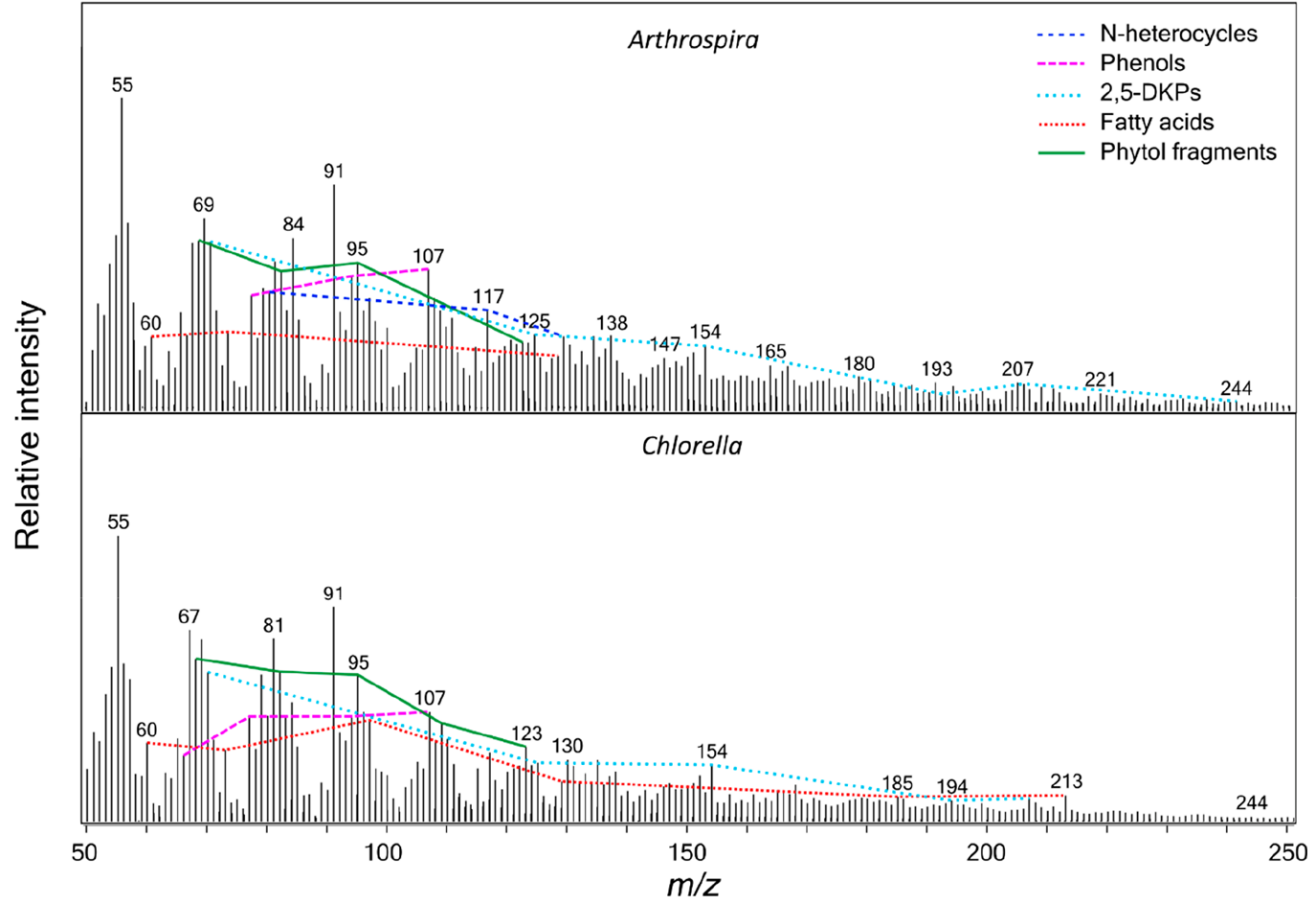
Pyrolysis–GC–MS MASPEX simulated mass spectra of *Arthrospira* and *Chlorella*. This data previously appeared in Salter et al.^42^ Ions from the same compound family are linked by lines, labeled according to the legend.

Figure 5 shows the MASPEX simulated mass spectra for the pyrolysis–GC–MS analysis of *Arthrospira* and *Chlorella*, using data from Salter et al.^42^ Comparing Figures 4 and 5 we can observe the effects of the hydrous component of processing. There are some similarities between the spectra with the dominant peaks (*m*/*z* 55, 67, 69, 91, 91, 107) being the same for the microorganisms that have undergone hydrous pyrolysis and extraction and for those analyzed by pyrolysis–GC–MS. However, other patterns are different, such as the presence of the alkylamide series (*m*/*z* 57, 73, 115, 129) in the hydrous pyrolysis data, which has a very low relative intensity in the pyrolysis–GC–MS spectra. The saturated fatty acid ion series (*m*/*z* 60, 73, 129, 157, 185, 213, 241, 256, 284) also has a higher relative intensity in the hydrous pyrolysis data compared to the pyrolysis–GC–MS data. Several ion series that are present in the pyrolysis–GC–MS data are absent from the hydrous pyrolysis data; these are the phytol fragments (*m*/*z* 68, 82, 95, 109, 123) and 2,5-diketopiperazines ion series (*m*/*z* 70, 125, 154, 194, 208, 244). The differences observed between the mass spectra from the two different analysis methods show how mass spectrometry can be used to distinguish the processes through which microorganisms have been subjected. These differences are due to the different mechanisms of the two techniques: pyrolysis–GC–MS is a rapid online anhydrous pyrolysis technique that thermally dissociates high molecular weight organic networks producing GC-amenable fragments that provide an insight into the chemical structure of the parent, whereas for hydrous pyrolysis, samples are subjected to hydrothermal conditions for longer durations.^65^

### Scientific Implications

The upcoming mission to Europa, with NASA’s Europa Clipper, will contain the mass spectrometer MASPEX. This powerful chemical analysis instrument will be able to detect organic molecules, and it has been constructed with the intention of being able to detect complex organic molecules indicative of habitability. In our previous work, we demonstrated that it is possible to use MASPEX to discriminate molecules that originate from a biotic source, particularly microorganisms that may be present on icy moons, to those from abiotic sources.^42^ In this paper, we have continued our analysis of microorganisms to show that the fragments of life from hydrothermally altered environments can be detected and differentiated from abiotic sources.

It has been shown previously that complex macromolecular organic compounds have been detected at the icy moon Enceladus and may be derived from a hydrothermal source.^15,66^ Here on Earth, hydrothermal environments are habitable and are thought to be one of the places where life originated.^16^ Therefore, it is plausible that life may have originated in a similar way on icy moons. Any organic remnants of life would be altered in hydrothermal environments, and that processing must be understood for effective interpretation of organic materials detected by mass spectrometry in the plumes of icy moons. Here, we have shown that the molecular architectures of microorganisms are altered by hydrothermal environments but that their diagnostic molecules can still be detected and differentiated from abiotic counterparts.

## Conclusions

We have shown that hydrothermal processing of the microorganisms, *Arthrospira* and *Chlorella*, using hydrous pyrolysis, leads to the degradation of bacterial components; however, some of the biological fingerprint remains at the highest experimental conditions of 300 °C held for 72 h. The biological signals consist of straight-chain even numbered saturated fatty acids, which are lipid fragments, as well as one-ring and two-ring aromatic compounds such as phenol and indole derivatives from protein and carbohydrate components. Intermediary pyrolysis products are also detected; these are alkylamides that are formed from condensation reactions between lipid and protein components.

The preservation of these diagnostic molecules, even under harsh alteration processes, provides confidence that they could be used as molecular fingerprints for the detection of biological materials on icy moons, should they exist there. Mass spectra were generated from the GC–MS analysis of samples after hydrous pyrolysis, and characteristic ions from the different components were identified. These spectra and ion series add to the mass spectral library that will be comparable with mass spectra and data obtained by MASPEX onboard the upcoming Europa Clipper mission, enabling the detection and identification of degraded lifeforms should they be present on icy moons.
